# Editorial: Immune responses to MTB infection in people living with HIV

**DOI:** 10.3389/fimmu.2024.1523101

**Published:** 2024-11-29

**Authors:** Elsa Anes, Wondwossen Amogne

**Affiliations:** ^1^ Research Institute for Medicines (iMed.ULisboa), Faculty of Pharmacy, Universidade de Lisboa, Lisboa, Portugal; ^2^ College of Health Sciences, School of Medicine, Department of Internal Medicine, Addis Ababa University, Addis Ababa, Ethiopia

**Keywords:** HIV, tuberculosis, co-infection, people living with HIV, immune cells, granuloma, exosomes, metabolism

Tuberculosis (TB) has once again become the world’s leading cause of death from a single infectious agent following three years of COVID19 pandemic (1). Accounting for 1.25 million deaths, TB remains the leading cause of death among people living with human immunodeficiency virus (HIV) (PLWH) (1), and was responsible for approximately twice as many deaths as HIV/acquired immunodeficiency syndrome (AIDS) alone (2). The immune responses to monoinfection with the causative agent, *Mycobacterium tuberculosis* (Mtb), are distinct from those observed in individuals infected with HIV or in those who are coinfected with both pathogens (3, 4). Pulmonary TB represents the most common manifestation of the active disease, characterized by the presence of classical lung caseous/necrotic granuloma lesions, accompanied by exudation. The disease typically progresses after two years of a latent TB infection, an asymptomatic immune activated phase following the initial primary infection (1, 5).

The immune cells involved in the responses to Mtb infection include innate myeloid cells, such as macrophages and neutrophils, and adaptive lymphocytes including T and B cells. If the equilibrium between innate and adaptive immune cells, which is essential for maintaining an appropriate proinflammatory/anti-inflammatory cytokine milieu, is disrupted, Mtb may disseminate from lungs to other organs, leading to the development of extrapulmonary TB. An appropriate adaptive response is necessary to facilitate the homing of effector T-lymphocytes, which is essential for the full organization and sustainability of the granuloma. This means that mycobacteria-containing macrophages are situated at the center, encircled by a rim of newly arrived lymphocytes that form a solid granuloma and exhibit a highly organized framework with high vascularization (6). A failure to maintain this dynamic will result in structural disruption, thereby facilitating the migration of infected macrophages to other organs (7).

It has long been established that functional impairments in both the innate and adaptive immune responses in PLWH contribute to an increased risk of TB disease in this population (8). As a consequence of the immunodeficiency status generated by low CD4^+^ T-cell counts, this population is also more prone to the development of independent or concurrent extrapulmonary TB. Consequently, the pathophysiology of Mtb infection differs in HIV-positive individuals, and even more so in those who have progressed to severe AIDS phase. The mechanisms by which alterations in Mtb-specific T-cell responses may impede the early clearance of Mtb or sustained control of LTBI in PLWH remain poorly understood. PLWH appear to display an altered cytokine profile in response to Mtb, which includes impairments in type I helper T- cell responses (Th1), a reduction in the activity of Th17 cells and regulatory T-cells, and an increase in immunosuppressive cytokines (8, 9). Furthermore, the incidence of smear-negative and subclinical TB is also higher among co-infected patients. This results in a reduction of the sensitivity of screening tests based on sputum examination.

The identification of immune-related complexes or molecules that can be defined as diagnostic markers for Mtb in PLWH may facilitate the development of new diagnostic tools and the exploration of new therapeutic approaches. In comparison to monoinfections with either HIV or Mtb, during coinfection the immune cells subsets and functions in the peripheral blood, as well as their redistribution in local lesions, may undergo dramatic alterations. Additionally, deviations in the metabolism and the divergence of the ubiquitin machinery may also be observed. Altogether, the aforementioned factors represent the focus of this Research Topic.

In this Research Topic, an original research article by Yandrapally et al. shows that the Mtb-secreted transcription regulator EspR contributes to a syndemic interaction during co-infection with HIV. It is hypothesized that binding to the putative cognate motif on the promoter region of the host IL-4 gene, leads to IL-4 gene expression, causing high IL-4 titers that induce a Th2-type microenvironment. Consequently, this results in a shift towards a Th2-type response, which facilitates macrophage polarization to a M2 anti-inflammatory and metabolic status that favors Mtb persistence. Chronic infection by HIV tends to induce a shift in the viral population from the R5 to the X4 phenotype, which is accompanied by an increasing in the expression of the CXCR4 receptor. This suggests that the mycobacteria-induced selection of X4 viruses may also be mediated by alterations in IL-4 levels, thereby favoring an enhanced HIV propagation.

A systematic review and meta-analysis by Xie et al. investigated the impact of vitamin D deficiency on the increased risk of HIV-infected individuals to develop active tuberculosis, compared to those with latent tuberculosis infection. Overall, the meta-analysis findings indicated that there were no significant variations in vitamin D levels between HIV-infected individuals, TB-infected individuals, and HIV-TB co-infected individuals. The prevalence of vitamin D deficiency was higher in the HIV-TB group than in the HIV group. Additionally, the administration of vitamin D supplements did not result in a discernible impact on CD4^+^ count and viral load in the HIV-infected group. Likewise, vitamin D had no impact on the time to sputum smear conversion, time to culture conversion, relapse, or mortality in the TB group.

In a further original study, Marsile-Medun et al. investigated the heterogeneity of granulocyte populations and the potential differences in phenotype and immunomodulatory capacity between low-density granulocytes (LDG) and normal-density granulocytes (NDG) in PLWH. They identified several surface markers that were differentially expressed between these two subsets, thus allowing their distinction and providing new insights into the properties of LDG in PLWH. Given that during HIV-1 infection and the AIDS-related pathological context, LDG have been associated with the severity of the infection and may have an immunosuppressive role through the release of arginase-1 and through PD-1/PD-L1 interactions with T cells, they may provide insights into the status of the infection.

Finally, the review by Habib et al. highlights the importance of exosomes in the context of HIV-1 pathogenesis, emphasizing the growing body of research exploring their potential as a therapeutic approach for achieving HIV-1 remission in PLWH. Engineered exosomes could be used as delivery systems for therapeutic molecules, including exosome-based HIV-1 vaccines. One of the key advantages is the regulated bio-delivery, which is enabled by their capacity to circulate throughout the body and to be effectively taken up by antigen presenting cells. In addition, different studies have shown that exosomes derived from breast milk and semen possess anti-HIV properties, suggesting that exosomes isolated from different biological sources in infected individuals could be used to assess HIV-1 disease progression. Further insights into the function of these immunomodulatory nanovesicles in the pathogenesis of AIDS will emerge from the identification of the biomolecules carried by exosomes and the clarification of their impact on immune regulation in PLWH.

The tenet of this Research Topic is that a better understanding of these issues will facilitate the development of novel diagnostic tools for the assessment of infection status, as well as therapeutic interventions to control both infections and mitigate deleterious inflammation, ultimately benefiting the host.
